# Comparing delayed matching to sample with three variations of the training‐IRAP for establishing derived relations

**DOI:** 10.1002/jeab.70082

**Published:** 2026-01-08

**Authors:** Marcello Henrique Silvestre, Colin Harte, Denise Aparecida Passarelli, Júlio César de Rose

**Affiliations:** ^1^ Universidade Federal de São Carlos São Carlos Brazil; ^2^ Instituto Nacional de Ciência e Tecnologia sobre Comportamento, Cognição e Ensino Brazil; ^3^ Instituto Par – Ciências e Tecnologia de Comportamento São Paulo Brazil; ^4^ National University of Ireland Maynooth Ireland

**Keywords:** delayed MTS(2s), derived relations, training format, training‐IRAP, yield

## Abstract

A common method for studying derived relations is the matching‐to‐sample (MTS) preparation. However, certain aspects of its training format potentially hinder the emergence of new relations. The training version of the implicit relational assessment procedure (training‐IRAP) may present an alternative. Our primary objective involved comparing the effectiveness of delayed MTS(2s) and training‐IRAP procedures on participant yield. The secondary objective involved comparing mean number of training blocks per procedure. Given additional components in the standard training‐IRAP not found in MTS, changes were made to the former, producing the modified training‐IRAP and delayed modified training‐IRAP(2s). Sixty‐eight typically developing students participated in a between‐subjects design. Two classes comprising five abstract stimuli were employed. Yield was analyzed at three levels, 91.67, 83.33, and 79% correct responses, with at least 87.5% correct responses at baseline mixed‐block maintenance. All participants maintained baseline criterion during tests. At the three levels of analyses, the modified versions of training‐IRAP produced higher yield, followed by DMTS(2s) and then the standard training‐IRAP. Mean number of blocks to complete training phases was lowest for the delayed MTS(2s) and delayed modified training‐IRAP(2s) groups. Limitations and implications of the findings toward greater precision, scope, and depth in conceptual, experimental, and applied settings are discussed.

Language and cognition as symbolic behavior have been studied by behavior analysts largely through research on emergent or derived relations (Barnes‐Holmes et al., 2018; Perez et al., 2023; Sidman, 1994). The most common procedure employed for this (i.e., establishing relations between stimuli and testing for new relations that were never directly taught) is matching to sample (MTS; Fienup & Brodsky, 2020; Pilgrim, 2020; Sidman, 1971). In a typical MTS preparation, a sample stimulus (e.g., A1) is presented, a response to which (e.g., clicking on that stimulus with a computer mouse) leads to the appearance of two or more comparison stimuli, one of which is experimentally correct (S+; e.g., B1) and the others incorrect (S−; e.g., B2). In the presence of A1, choosing B1 is reinforced, whereas choosing B2 results in no programmed reinforcement. The response to B2 will only be reinforced if presented with an alternative sample stimulus, A2. Given the training of AB and BC relations (two trained relations), the learner typically demonstrates, without direct training, the derived relations BA, CB, AC, and CA (four untrained, derived relations).

Although various facilitating parameters for the emergence of derived relations have been studied within the MTS procedure (Arntzen, 2012), some aspects of MTS have been shown to hinder the emergence of new relations and transfers of stimulus function (i.e., a phenomenon whereby a stimulus acquires the functions of another stimulus with which it has been related; e.g., see Dougher et al., 1994; Martins et al., 2023; Perez, de Almeida, et al., 2020). These aspects include different sources of stimulus control during baseline training (i.e., selection and rejection control; e.g., Carrigan & Sidman, 1992; Johnson & Sidman, 1993; Perez et al., 2015, 2017; Perez, Vaidya, et al., 2019; Perez, Huziwara, et al., 2020). In the example above, choosing B1 can be controlled by both the relation between the sample stimulus (i.e., A1) and the comparison stimulus defined as S+ (i.e., B1) and the relation between the sample stimulus and the comparison stimulus defined as S− (i.e., B2). In the latter case, B2 is argued to control the selection of the other available comparison stimulus (i.e., B1). In other words, the individual would reject B2 and select B1. Thus, the choice response may be controlled by both the sample‐S+ relation (selection control), where the individual responds to S+, and/or the sample‐S− relation (rejection control), where the individual responds to S− (de Rose, 1996). However, the controlling relation may not be evident in any given case without additional tests (Perez & Tomanari, 2013).

Despite the initial hypothesis that rejection control would hinder tests of derived relations (Carrigan & Sidman, 1992), some studies have demonstrated better performance on derived‐relations tests when conjoint S+ and S− control is established for all baseline relations (e.g., Arantes & de Rose, 2015; de Rose et al., 2013; Plazas, 2019). In addition, other studies have indicated the importance of S− control for producing derived relations (Harrison & Green, 1990; Plazas & Peña, 2016; Plazas & Villamil, 2016a, 2016b). However, to induce control by both S+ and S− in an MTS task, additional procedures are necessary, and their effectiveness often depends on combinations of procedures (see Perez & Tomanari, 2014, for a methodological review; see also Perez, Vaidya, et al., 2019, for an empirical analysis of procedures used to establish S− control). On the other hand, some studies, following Carrigan and Sidman's hypothesis, have moved beyond the two‐choice stimulus comparison in the MTS procedure to avoid S− control and increase the likelihood of S+ control—for example, introducing three or more choices (e.g., Dias et al., 2021; Fields et al., 2012; see also Boldrin et al., 2022, for the two‐choice MTS procedure with rotating S− stimuli).

The training‐IRAP, a procedure originating from relational frame theory (RFT; Barnes‐Holmes & Harte, 2022a; Hayes et al., 2001), has been employed recently to establish relations between stimuli and testing for derived relations (e.g., Harte et al., 2018, 2020, 2021; Leech & Barnes‐Holmes, 2020; Leech et al., 2018). This method is a version of the implicit relational assessment procedure (IRAP; Barnes‐Holmes et al., 2010), a task originally developed to assess the probability of particular relational responses. However, whereas the IRAP procedure requires that participants alternate between responses consistent with and contrary to their individual learning histories (i.e., to assess response probability), the training‐IRAP does not require such contrary responding (i.e., it is simply used to train particular relations; see Barnes‐Holmes & Harte, 2022b, for more details on the difference between the training‐IRAP and IRAP format). The training‐IRAP procedure involves presenting a pair of stimuli (e.g., A1 and B1 or A1 and B2) and two response options (e.g., “Same” and “Different”). The task requires that participants respond both quickly and accurately to the stimulus pair with one of the two response options.^1^

With respect to training trial format, MTS and the training‐IRAP differ. In an MTS trial, the participant selects a comparison stimulus that “goes with” the sample. For example, given A1 as the sample and B1 (S+) and B2 (S−) as comparisons, the trial assesses whether the participant selects B1 when presented with A1 (i.e., whether A1 goes with B1). In essence, the response involved is the choosing of the stimulus. In contrast, the training‐IRAP specifies the relations (Same or Different) between the sample stimulus and each comparison (S+ and S−) individually. For example, A1 and B1 (S+) are designated as Same, and A1 and B2 (S−) as Different, thereby evaluating the stimulus relation between the sample and both comparison S+ and S− stimuli. In summary, whereas MTS assesses Sample/S+, the training‐IRAP assesses, individually, Sample/S+ (i.e., Same) and Sample/S− (i.e., Different).

The primary objective of the present study was to compare the effectiveness of MTS and training‐IRAP procedures with respect to subsequent tests for derived relations. The primary measure used was *yield* (Fields et al., 2020), a commonly employed outcome in studies of class formation (e.g., Ayres‐Pereira & Arntzen, 2021; Hensel et al., 2024; Marin et al., 2022), defined as the percentage of participants who reached the required criterion for class formation (i.e., a set of stimuli that have come to function interchangeably).

The secondary objective was to compare the mean number of training blocks needed per procedure. With respect to MTS, we used the delayed matching‐to‐sample 2‐s [DMTS(2s)] format rather than other alternatives because this preparation has typically proven to be particularly effective for increasing the likelihood of class formation relative to simultaneous matching‐to‐sample (e.g., Arntzen, 2006; Arntzen et al., 2014, 2015; Bortoloti & de Rose, 2009, 2012; Ribeiro & de Souza, 2024; Vaidya & Smith, 2006).

Overall, our main rationale was to explore the influence of different task training formats on the emergence of derived relations. In addition, because the training‐IRAP (hereafter referred to as *standard training‐IRAP*) comprises additional components not usually found in MTS, a modified version, excluding these components, was created and also employed: the *modified training‐IRAP*. This modification allowed for a methodological approach more comparable to that of the DMTS(2s) while still encompassing the main differences in the training formats. Specifically, the modified training‐IRAP removed the requirement to respond under time pressure, changed the response topography from keyboard press to mouse click, eliminated the correction procedure, and excluded postblock accuracy information (see Method below for a full explanation). Upon further reflection, we noted two additional DMTS(2s) components known to positively affect yield but that were not involved in the standard training‐ or modified training‐IRAPs, specifically, the observational response to the stimuli and the 2‐s delay between the removal of the sample stimulus and the presentation of the comparison stimuli. Consequently, to make the modified training‐IRAP more structurally similar to the DMTS(2s) and investigate the influence of these variables on training and testing for derived relations, these final alterations were made to the modified training‐IRAP, making it a delayed modified training‐IRAP(2s).

Therefore, the study comprised four procedures that were compared in terms of yield and mean number of blocks to establish baseline relations: (1) DMTS(2s), (2) standard training‐IRAP, (3) modified training‐IRAP, and (4) delayed modified training‐IRAP(2s).

## METHOD

### Participants

Sixty‐eight students, from the Federal University of São Carlos, participated (males = 36, females = 32), with an age range of 18–30 years (*M* = 21, *SD* = 2.6). They were native speakers of Brazilian Portuguese, without any mental health diagnosis, without knowledge of stimulus equivalence and RFT, and without prior experience with the procedures involved in this study. The participants were recruited through convenience sampling. Before the experiment, they read and signed an informed consent form (approved by the Brazilian platform for ethical committees, Plataforma Brasil, CAAE: 56710222.0.0000.5504). At the end of the experiment, participants were fully debriefed and received no compensation for participation.

### Location and equipment

The study was conducted in an experimental cubicle at the Federal University of São Carlos using a computer (LG, Intel Core i5‐5200U CPU @ 2.20GHz, 4G RAM, 1 TB HD) equipped with the PsychoPy software (Peirce et al., 2019). Procedures were programmed with this software, which automatically recorded and stored data in an Excel spreadsheet.

### Stimuli

**FIGURE 1:**
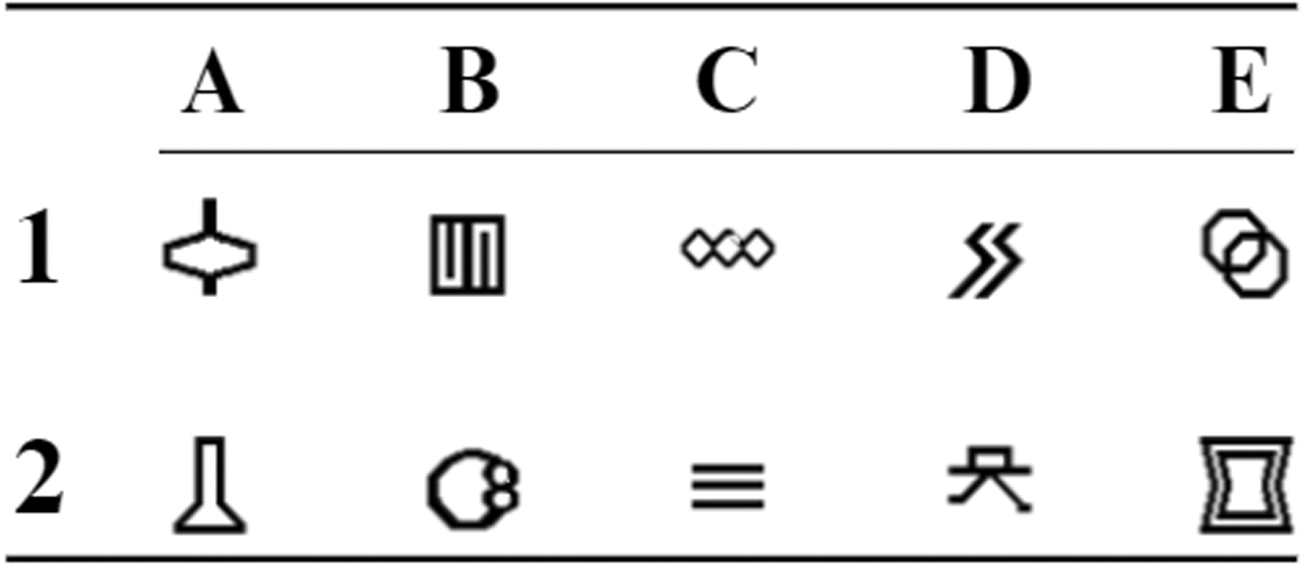
Stimuli used in the experiment. Each stimulus is labeled with a number and a letter. Numbers indicate the class (i.e., Class 1 and Class 2), and letters indicate the exemplar within that class.

Ten abstract shape stimuli were used in the study (see Figure 1) that align with those previously employed in the literature (e.g., de Almeida & de Rose, 2015; Gomes et al., 2019; Mizael et al., 2021; Silveira et al., 2021). While it is possible that the specific stimuli influenced the degree to which classes were formed, it is important to emphasize that the same stimuli were used consistently across all procedures, enabling meaningful comparisons. Additionally, no participant had prior experience with experimental psychology research, increasing the likelihood that all stimuli were novel to them. Nevertheless, asking participants to evaluate the stimuli before the experiment may be a worthwhile practice to incorporate in future related research (see Hensel et al., 2024).

### Experimental design

We employed a between‐groups comparison design. Participants were equally and randomly divided into four experimental groups, each with *N* = 17: DMTS(2s), standard training‐IRAP, modified training‐IRAP, and delayed modified training‐IRAP(2s).

### Dependent variables

The main comparison between groups was the number of participants who reached the preexperimentally established criteria (see below) for class formation (yield). The groups were also compared based on the mean number of blocks needed to complete the baseline relation training.

### Procedure

The experimental session took place in a single day and involved relational training intended to establish two 5‐member stimulus classes: A1B1C1D1E1 and A2B2C2D2E2. A linear series (LS) training structure was chosen to avoid a ceiling effect (Arntzen, 2012; Imam, 2006). After baseline training, baseline mixed training blocks were presented, first with differential consequences and then without. Following the attainment of all criteria, probes for derived relations were conducted using a complex‐to‐simple (CTS) protocol (cf. Imam, 2006). This protocol involved testing from the largest to the smallest nodal^2^ distance (i.e., EA, DA, EB, CA, and DB, respectively) divided into blocks and interspersed within baseline mixed training blocks without differential consequences. Criteria for class formation were analyzed at three levels of stringency: a minimum score on each probed relation of (1) 91.67% (22/24) correct responses, (2) 83.33% (20/24) correct responses, and (3) 79% (19/24) correct responses. Participants also needed to achieve at least 87.5% (14/16) correct responses in the baseline mixed training blocks interspersed between the probes. These criteria were applied to all groups.

The yield criterion, set at three different levels (91.67, 83.33, and 79%), was chosen to address the complexity of the probe blocks, which included five separate probe derived relations: EA, DA, EB, CA, and DB. This approach ensured a minimum standard score for each probed relation. Class formation was confirmed only if participants met the criterion for each probed relation and also maintained the 87.5% criterion in the mixed baseline blocks interspersed between probes. Analyzing three levels of criteria allowed for the evaluation of the number of participants who met these preestablished standards, ranging from the most to the least strict.

In three of the four training and testing procedures—DMTS(2s), modified training‐IRAP, delayed modified training‐IRAP(2s)—participants selected stimuli by clicking on them with the mouse. In contrast, the training and testing procedures involved in the standard training‐IRAP required pressing specific keys on the computer keyboard to respond to stimuli. The differences in response topographies (mouse vs. keyboard) arose because the standard training‐IRAP format typically employs the keyboard, whereas the modified versions were designed to incorporate the mouse to better align with response topographies typical of MTS studies (see Kato et al., 2008, for how different response topographies influence class formation).

#### DMTS(2s)

**FIGURE 2:**
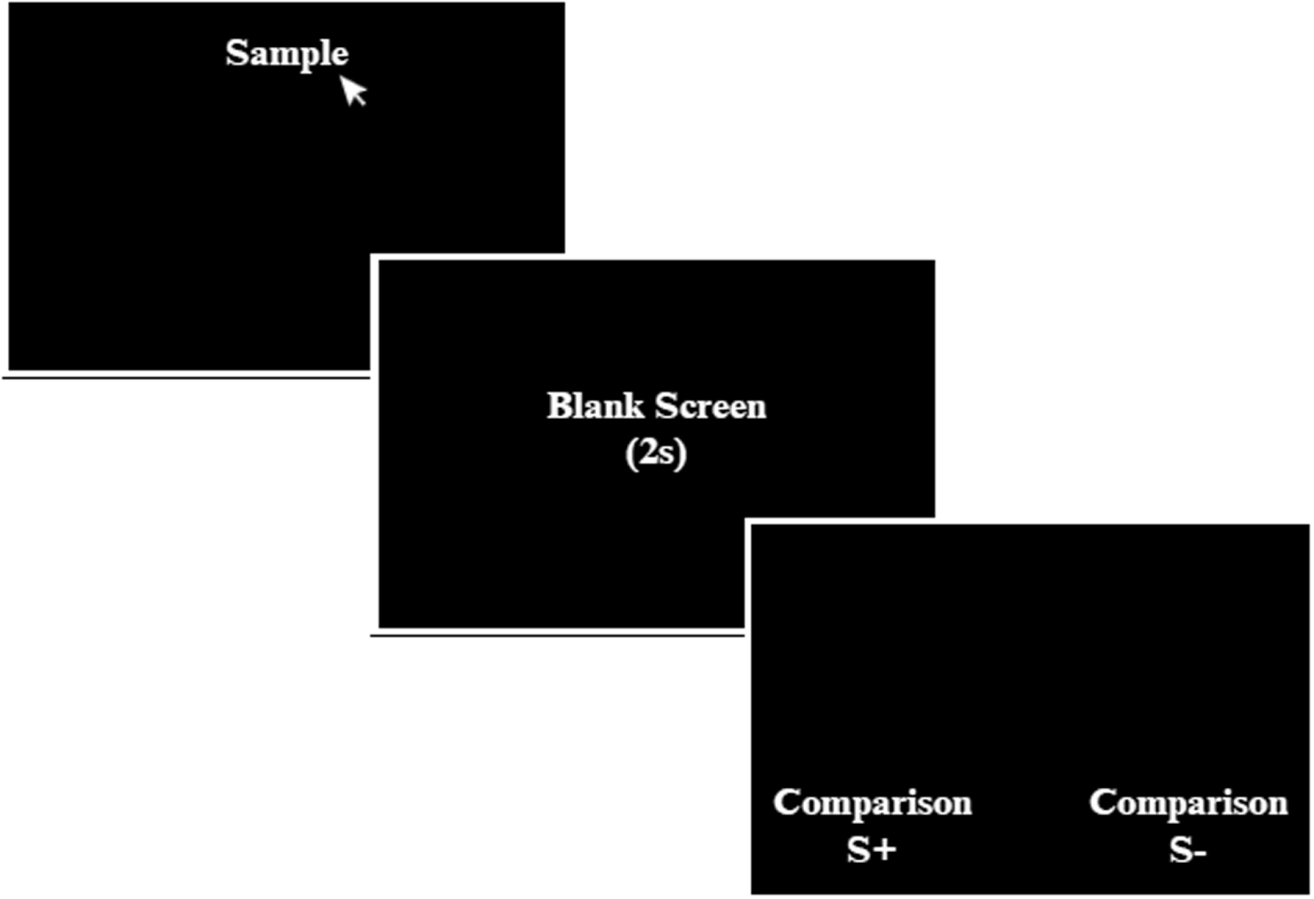
Training format of delayed matching‐to‐sample (2s). In each trial, one of the sample stimuli was presented. An observational response to the sample was conducted by clicking on that stimulus with a computer mouse. The blank screen was represented by a completely black screen and lasted 2s. The positions of the stimulus comparisons (i.e., the experimentally correct S+ or the incorrect S−) were randomized across trials. The selection of one of the comparisons was made by clicking on the stimulus with a computer mouse.

Each trial started with the presentation of a sample stimulus (e.g., A1) at the top of the screen. An observing selection response to the sample produced its withdrawal, and after 2s, two comparison stimuli (e.g., B1 and B2) were displayed side by side at the bottom of the screen (see Figure 2). Selecting one resulted in the withdrawal of all, proceeded by one of the following differential consequences presented for 1 s: A correct response (e.g., selecting B1 given sample A1) produced the word “Correct” in green, and an incorrect response (selecting B2 given sample A1) produced the word “Wrong” in red. The screen was then darkened in an intertrial interval (ITI) of 40 ms, which separated the end of the consequence from the beginning of the next trial. In an AB training block, the training format comprised two types of trials: A1/B1B2 and A2/B1B2. The stimulus before the slash (/) indicates the sample, and the stimuli after the slash indicate the comparisons, with the underlined stimulus representing the comparison defined as correct (i.e., Sample/Comparisons S+ and S−). In other words, if Sample A1, then choose B1; if Sample A2, then choose B2.

##### DMTS(2s) instructions

Before the start of the experiment, written instructions were provided (in Portuguese), and if there were no questions, the experiment began. The following instructions were provided for all groups: “*Please, store your cellphone (put it on silent) and avoid using it during the experimental session*.” and “*There will come a point in the experiment where the computer will no longer indicate if choices are correct or incorrect, but there will always be a correct response, and the computer will continue to record whether the choice was correct or not. If you have any questions, ask the experimenter*.” Specific instructions for the DMTS(2s) group were as follows:

*Initially, an image will be presented at the top of the screen. Observe the image and click on it with the left mouse button. This image will disappear, and two other images will appear shortly afterward at the bottom of the screen. Your task is to discover the combinations/relations of these images with the one that appeared at the top of the screen. To select one of the images, move the mouse cursor to it and click the left mouse button. If your choice is correct, the word “Correct” (in green) will appear on the screen, and if your choice is incorrect, the word “Wrong” (in red) will appear in the center of the screen*.

#### Standard training‐IRAP

**FIGURE 3:**
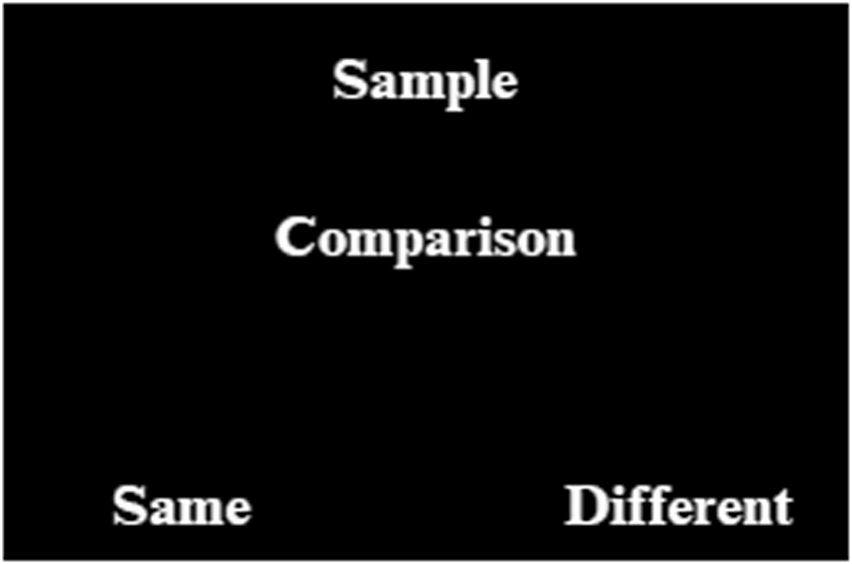
Training format of standard training‐IRAP. In each trial, one of the sample stimuli was presented along with one of the comparison stimuli and two response options (i.e., “Same” and “Different”) with their positions randomized between trials. The selection of one of the response options was made using the keyboard. The “D” key on the keyboard was used to select the response option on the left, and the “K” key was used to select the option on the right.

Each trial began with the presentation of a sample stimulus at the top of the screen (e.g., A1), a comparison stimulus in the center of the screen (e.g., B1), and two response options at the bottom corners of the screen (i.e., “Same” and “Different”), with their positions randomized for each trial^3^ (see Figure 3). Participants pressed the “D” key on the keyboard to select the response option on the left and the “K” key to select the option on the right. If the chosen option was correct (e.g., if the response option “Same” was selected on presentation of sample A1 and comparison B1), the word “Correct” was displayed in green for 1 s, positioned on the screen between the response options. If the chosen response was incorrect (e.g., if in response to A1 and B1, “Different” was selected), a red X appeared between the response options and remained until the correct response option (i.e., “Same”) was selected. After delivering the feedback “Correct” or following the correction of the incorrect response, there was an ITI of 40 ms before the start of a new trial. At the end of each block of trials, the participant was presented with a screen displaying their percentage of correct responses and response latency for that block. This screen remained visible until the participant pressed the spacebar, as indicated by the instruction at the bottom of the screen: “Press spacebar to continue.” Additionally, the scores (accuracy and latency) required to advance to the next block were shown. A mean latency ≤ 3,000 ms was required. If necessary, a performance‐specific tip was provided (e.g., “Respond with greater accuracy,” “Respond more quickly.”). Unlike MTS, the training‐IRAP format training blocks consisted of four types of trials based on the sample–comparison stimulus combinations. For example, in the AB training block, the training trial format was A1/B1/Same‐Different, A1/B2/Same‐Different, A2/B1/Same‐Different, and A2/B2/Same‐Different. The stimulus before the first slash (/) indicates the sample, the stimulus before the second slash indicates the comparison, and “Same” and “Different” are the response options provided, with the underlined response option indicating the correct choice (i.e., Sample/Comparison S+ or S−/Same–Different).

##### Standard training‐IRAP instructions

*You will need to select one of the words “Same” and “Different” that will be presented at the bottom of the computer screen. An image will be shown at the top of the screen, followed by another one presented at the center. Your task is to discover the relationship between the images by selecting one of the options, “Same” or “Different,” whose positions will be randomized in each trial. To choose the response option on the left side of the screen, press the “D” key, and to select the option on the right, press the “K” key on your computer. If your choice is correct, the word “Correct” (in green) will appear on the screen and you will move on to the next trial. If your choice is incorrect, an “X” (in red) will appear on the screen. To proceed to the next trial, select the correct response option. At the end of each block of trials, a screen will show your percentage of correct responses and speed in the block, along with the scores you need to achieve to advance in the task. If necessary, a hint (in red) will also appear. Respond with the utmost accuracy. When you learn to be precise, you will naturally respond faster as well*.

#### Modified training‐IRAP

The trial format of this procedure remained the same as the standard training‐IRAP described above. However, some components were changed or removed with the goal of making it more similar to MTS: (1) The selection of one of the response options was now made using the mouse (i.e., clicking with the mouse on one of the response options) instead of pressing the “D” and “K” keys on the keyboard. (2) The correction requirement following an incorrect response was removed. Thus, after emitting an incorrect response, the feedback “Wrong” was displayed in red for 1 s and the trial ended. (3) The latency criterion for making a response was removed, as was (4) the postblock screen displaying that block's accuracy and latency.

##### Modified training‐IRAP instructions

As noted above, in the modified versions of the training‐IRAP, the typical score screen (accuracy and latency) was not presented at the end of each block of trials in an attempt to reduce additional extraneous variables not present in MTS studies. In addition, although instructions for the modified training‐IRAP group were otherwise the same as the previous group, the following two modifications were made regarding (a) making a response and (b) feedback, respectively:

*To select one of the response options (“Same” or “Different”), move the mouse cursor over the desired option and click the left mouse button. If your choice is correct, the word “Correct” (in green) will appear on the screen, and if your choice is incorrect, the word “Wrong” (in red) will appear in the center of the screen*.

#### Delayed modified training‐IRAP(2s)

**FIGURE 4:**
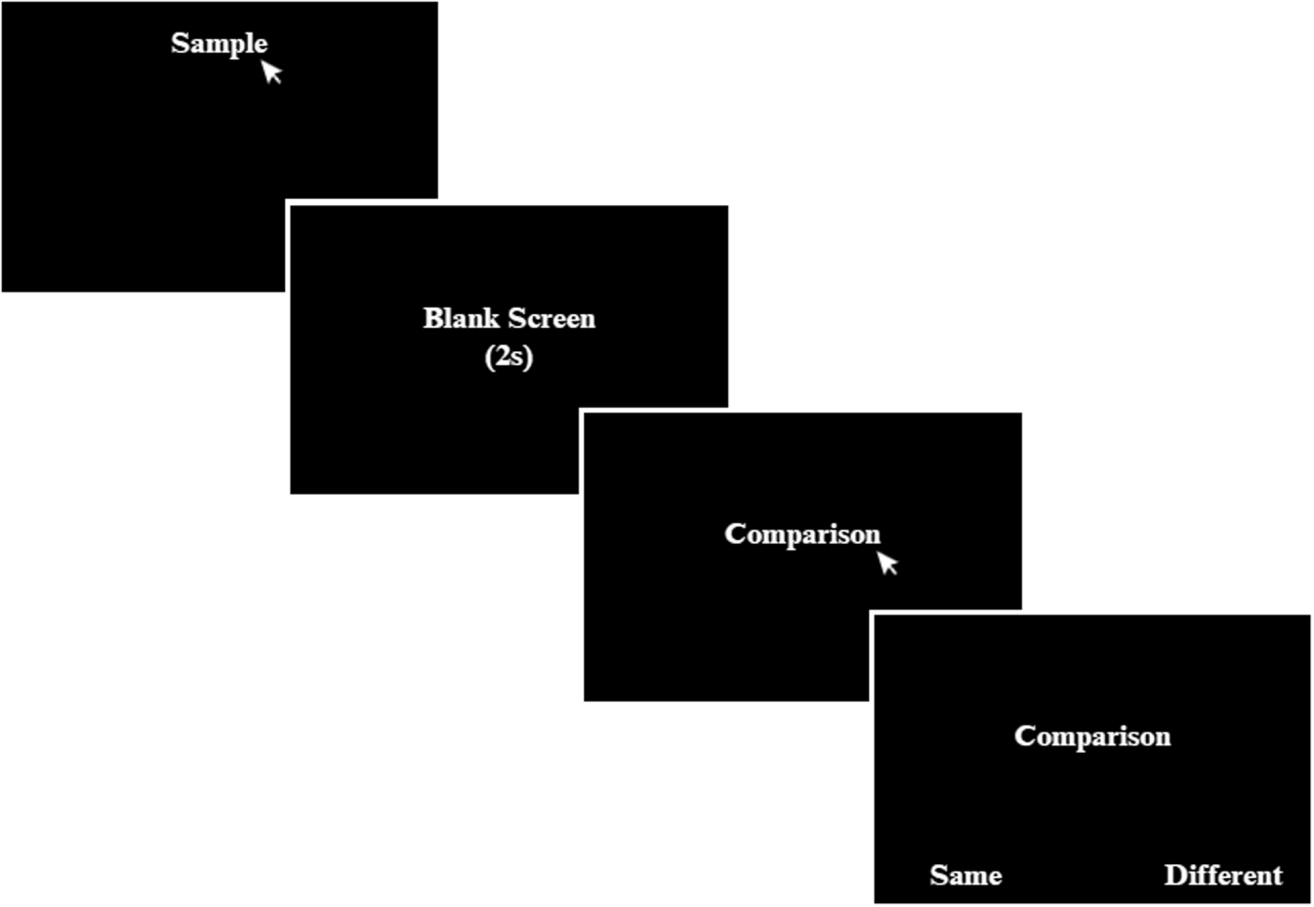
Training format of delayed modified training‐IRAP (2s). In each trial, one of the sample stimuli was presented. An observational response to the sample was conducted by clicking on that stimulus with a computer mouse. The blank screen was represented by a completely black screen and lasted 2s. One of the comparison stimuli was then presented, an observational response to which was required to proceed. After the observational response, the comparison stimulus remained on the screen and two response options (i.e., “Same” and “Different”) with their positions randomized across trials were immediately presented. The selection of one of the response options was made by clicking on an option with a computer mouse.

This procedure was identical to that for the modified training‐IRAP, except that the 2‐s delay component present in DMTS(2s) was added. Thus, a trial began with the presentation of the sample (e.g., A1). After an observing response to the sample was emitted, this stimulus was removed and, after 2s, one of the comparison stimuli (e.g., B1 or B2) appeared. An observing response to the comparison stimulus was also required, followed immediately by the presentation of the two response options: “Same” and “Different” (see Figure 4).

##### Delayed modified training‐IRAP(2s) instructions

The instructions involved for the delayed modified training‐IRAP(2s) were the same as for the previous group with one exception. Specifically, to reflect the additional observational response and 2‐s delay components involved, the initial part of the instructions was modified:

*Initially, an image will be presented at the top of the screen. Observe the image and click on it with the left mouse button. This image will disappear, and another image will appear shortly afterward in the center of the screen. Observe the image and click on it with the left mouse button. Right after, two words will appear at the bottom of the screen: “Same” and “Different”*.

The rest of the instructions were identical to those for the modified training‐IRAP group.

Table 1 summarizes the main features of the four procedures, indicating similarities and differences contained therein.

**TABLE 1:** Summary of the main features in the four experimental procedures.

Feature	DMTS (2s)	Standard training‐IRAP	Modified training‐IRAP	Delayed modified training‐IRAP (2s)
**Trial format**	Sample, S+, S−	Sample, S+ or S−, Same/Different	Sample, S+ or S−, Same/Different	Sample, S+ or S−, Same/Different
**Response topography**	Mouse click	Key press	Mouse click	Mouse click
**Feedback for incorrect responses**	Red “Wrong”	Red “X”	Red “Wrong”	Red “Wrong”
**Error correction / Block feedback**	No	Yes	No	No
**Baseline learning criteria**	Accuracy	Accuracy + Latency	Accuracy	Accuracy

#### Experimental phases

##### Phase 1: Baseline relation training

The training blocks were divided and respectively trained in the following order: AB, BC, CD, and DE, each block consisting of 24 trials. In the DMTS(2s) group, each sample type appeared in 12 trials per block (e.g., 12 trials of A1/B1B2 and 12 trials of A2/B1B2 in the AB training block). In the standard training‐IRAP, modified training‐IRAP, and delayed modified training‐IRAP(2s) groups, each trial type appeared six times per block (e.g., 6 trials of A1/B1/Same‐Different, 6 trials of A1/B2/Same‐Different, 6 trials of A2/B1/Same‐Different, and 6 trials of A2/B2/Same‐Different in the AB training block). The differential consequences for correct and incorrect responses were the words “Correct” and “Wrong” presented for 1 s. Participants were required to make 22 correct responses per block (>90% accuracy), and in the standard training‐IRAP, modified training‐IRAP, and delayed modified training‐IRAP(2s) groups, an additional criterion of no more than one error per trial type was employed to ensure a minimum of 83.3% correct responses for each trial type. In the standard training‐IRAP group, there was an additional requirement for mean response latencies to be less than or equal to 3,000 ms.

Before continuing, it is important to emphasize that although the same baseline mastery criterion (≥22/24 correct responses) was applied across all groups, some adjustments were required for the training‐IRAP groups due to the trial‐type configuration. For instance, in the DMTS(2s) group, an AB block consists of two trial types, each presented for 12 trials, within a total of 24 trials per block. In this configuration, making two errors within the same trial type does not significantly affect mastery of that specific trial type (i.e., achieving 10/12 correct responses corresponds to 83.33%). In contrast, the training‐IRAP procedures involve four distinct trial types within each AB block. Although the total number of trials per block remains 24, each trial type appears only six times. Consequently, the occurrence of two errors within the same trial type disproportionately affects mastery of that specific response (i.e., achieving 4/6 correct responses corresponds to 66.67%). To address this discrepancy and ensure consistent mastery across all trial types, an additional accuracy criterion of “no more than one error per trial type” was applied to the training‐IRAP groups. This corresponds to achieving at minimum 5/6 correct responses (83.33%) for each trial type.

##### Phase 2: Baseline mixed training

This block consisted of the randomized presentation of all previously trained baseline relations, with each block comprising 64 trials. Both the ITI and the presentation of differential consequences remained the same as in Phase 1. In the DMTS(2s) group, each sample appeared in eight trials (i.e., 8 trials of A1/B1B2, 8 trials of A2/B1B2, 8 trials of B1/C1C2, 8 trials of B2/C1C2, and so on). In the standard training‐IRAP, modified training‐IRAP, and delayed modified training‐IRAP(2s) groups, each trial type appeared in four trials (i.e., 4 trials of A1/B1/Same‐Different, 4 trials of A1/B2/Same‐Different, 4 trials of A2/B1/Same‐Different, 4 trials of A2/B2/Same‐Different, 4 trials of B1/C1/Same‐Different, 4 trials of B1/C2/Same‐Different, and so on). The required performance was 58 correct responses in the block (>90% accuracy). In the standard training‐IRAP, modified training‐IRAP, and delayed modified training‐IRAP(2s) groups, an additional criterion of no more than one error per trial type was set. Once again, ≤ 3,000‐ms mean response latencies were required for only the standard training‐IRAP group. If these criteria were not met but at least 75% of the responses were correct (i.e., 48 in total), the block could be repeated up to two more times. However, if any of the required criteria were not reached after three presentations of this block or if the total correct responses in any presentation fell below 75%, the participant returned to the previous phase.

##### Phase 3: Baseline mixed training without differential consequences

This block was identical to that for the previous phase, except that differential consequences were removed. If any of the same criteria as above were not achieved in the first presentation of the block, the participant returned to Phase 2.

##### Phase 4: Probe for derived relations

The probes for derived relations were divided into EA, DA, EB, CA, and DB blocks, in that order, with each block consisting of 24 trials. These were interspersed with baseline mixed training blocks, each comprising 16 trials. None of these blocks had differential consequences. In the DMTS(2s) group, each test block had 12 trials per sample (e.g., 12 trials of E1/A1A2 and 12 trials of E2/A1A2 in the EA test block). In the standard training‐IRAP, modified training‐IRAP, and delayed modified training‐IRAP(2s) groups, there were six trials per trial type (e.g., 6 trials of E1/A1/Same‐Different, 6 trials of E1/A2/Same‐Different, 6 trials of E2/A1/Same‐Different, 6 trials of E2/A2/Same‐Different, and so on). In the baseline mixed training blocks, interspersed between testing blocks, there were two trials per sample in the DMTS(2s). In the standard training‐IRAP, modified training‐IRAP, and delayed modified training‐IRAP(2s) groups there was one trial per trial type.

The sequence of experimental phases for all groups is summarized in Table 2.

**TABLE 2:** Sequence of experimental phases for all groups.

Phases	Relations	Trials per block	Accuracy criterion (*)	Percentage of consequences
Baseline training				
1.1	AB	24	22/24	100
1.2	BC	24	22/24	100
1.3	CD	24	22/24	100
1.4	DE	24	22/24	100
Baseline mixed training				
2	AB, BC, CD, DE	64	58/64	100
3	AB, BC, CD, DE	64	58/64	0
Probes for derived relations				
4.1.1	EA	24	(**)	0
4.1.2	AB, BC, CD, DE	16	14/16	0
4.2.1	DA	24	(**)	0
4.2.2	AB, BC, CD, DE	16	14/16	0
4.3.1	EB	24	(**)	0
4.3.2	AB, BC, CD, DE	16	14/16	0
4.4.1	CA	24	(**)	0
4.4.2	AB, BC, CD, DE	16	14/16	0
4.5.1	DB	24	(**)	0

*Note*: All training‐IRAP groups used an additional criterion of no more than one error per trial type. In the standard training‐IRAP group, an additional criterion was applied: Mean response latencies per block could not be longer than 3,000 ms.

* During the baseline training phases, the DMTS(2s) group used only an overall accuracy criterion.

** There was no accuracy criterion for the probes of derived relations. Accuracy was analyzed at three levels of stringency: 22/24, 20/24, and 19/24 correct responses.

## RESULTS

### Yield

**FIGURE 5:**
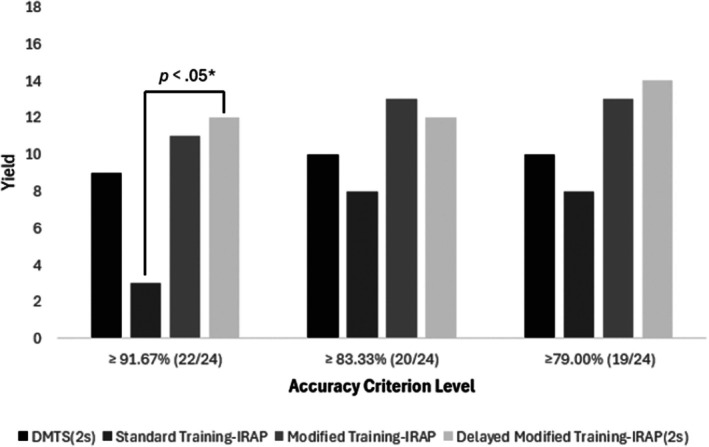
Yield per group at three levels of criterion stringency. Each bar represents the number of participants in each group who successfully met the class formation criteria, evaluated at three levels of stringency: a minimum of 91.67% (22/24), 83.33% (20/24), and 79% (19/24) correct responses.*Bonferroni‐adjusted *p* value.

The criteria for class formation were analyzed at three levels of stringency: a minimum score on each probed relation of (1) 91.67% (22/24) correct responses, (2) 83.33% (20/24) correct responses, and (3) 79% (19/24) correct responses. To meet each criterion, participants were also required to achieve at least 87.5% (14/16) correct responses in the baseline mixed training blocks interspersed between the probes. All 68 participants maintained the baseline criterion during the probe phase. Figure 5 presents the yield across the three levels of stringency for each group, showing the number of participants who met the criteria for class formation (for individual data, see Appendix A, Table A1).

Considering the DMTS(2s) group alone, one might infer the role of S− control for a number of individual participants. According to Carrigan and Sidman (1992), opposite results are expected in equivalence tests (as well as reflexivity and transitivity) with odd nodes when rejection control (i.e., S− control) is established during the baseline training. In the current preparation, EA, CA, and DB had an odd number of nodes: EA (3 nodes) and CA and DB (1 node each). Analysis of individual participant DMTS(2s) data (see Appendix A, Table A1) revealed that P03, P04, P10, P11, and P17 exhibited opposite results (i.e., accuracy ≤2/24) in odd numbers of nodes in the probed relations cited above (i.e., EA, CA, and DB). However, only P03, P11, and P17 demonstrated accuracy ≥23/24 for probed relations with even numbers of nodes (i.e., DA and EB). For P04 and P10, performance varied with an even number of nodes, respectively, 18/24 and 24/24 in the DA probed relation and 1/24 and 2/24 in the EB probed relation. In other words, P04 and P10 scored low in one probed relation with an even number of nodes (i.e., EB). Perhaps, therefore, S− control occurred during the baseline training for P03, P11, and P17, although this remains less conclusive for P04 and P10.

#### Yield based on the 91.67% (22/24) correct responses criterion

Based on the yield criterion of a minimum of 91.67% (22/24) correct responses on each probed relation, nine participants met criterion in the DMTS(2s) group, three in the standard training‐IRAP group, 11 in the modified training‐IRAP group, and 12 in the delayed modified training‐IRAP(2s) group. Given the small sample sizes of the groups, a Fisher's exact test (4 × 2) was conducted to assess the differences in yield between the groups (Fields et al., 2020). This test revealed a significant difference between the groups (two‐tailed *p* < .01). A post hoc Dunn test with Bonferroni‐adjusted contrasts was conducted, adjusting each *p* value by multiplying it by the number of comparisons (*p* × 6). With the *p* value adjusted with the Bonferroni correction, a significant difference was observed between the delayed modified training‐IRAP(2s) and standard training‐IRAP groups (*p* = .029, 95% CI [1.8–81.9]). The other group comparisons did not show statistically significant differences: DMTS(2s) × Standard Training‐IRAP (*p* = .42, 95% CI [0.9–37.2]), Modified Training‐IRAP × DMTS(2s; *p* = 1, 95% CI [0.3–8.1]), Delayed Modified Training‐IRAP(2s) × DMTS(2s; *p* = .1, 95% CI [0.4–11.2]), Modified Training‐IRAP × Standard Training‐IRAP (*p* = .06, 95% CI [1.4–61.3]), and Delayed Modified Training‐IRAP(2s) × Modified Training‐IRAP (*p* = 1, 95% CI [0.3–7.1]).

#### Yield based on the 83.33% (20/24) correct responses criterion

In line with the yield criterion of 83.33% (20/24) correct responses on each probed relation, 10 participants in the DMTS(2s) group, eight in the standard training‐IRAP group, 13 in the modified training‐IRAP group, and 12 in the delayed modified training‐IRAP(2s) group met criterion. The same statistical analysis was applied. Fisher's exact test indicated no significant difference between the groups (two‐tailed, *p* = .34).

#### Yield based on the 79% (19/24) correct responses criterion

In accordance with the yield criterion of 79% (19/24) correct responses on each probed relation, 10 participants in the DMTS(2s) group, eight in the standard training‐IRAP group, 13 in the modified training‐IRAP group, and 14 in the delayed modified training‐IRAP(2s) group met criterion. The same statistical analysis was applied. Fisher's exact test indicated no significant difference between the groups (two‐tailed, *p* = .13).

### Mean number of trial blocks to complete training phases

The mean number of blocks to complete the training phases (i.e., baseline training, baseline mixed training, and baseline mixed training without differential consequences) are presented in Table 3 (see Appendix B, Tables B1, B2, B3, B4, for complete individual data per block and procedure).

**TABLE 3:** Mean number of blocks to complete the training phases.

Procedure	Baseline training	Baseline mixed training	Baseline mixed training without differential consequences
DMTS(2s)	4.8	1.2	1.0
Standard training‐IRAP	11.2	2.7	1.5
Modified training‐IRAP	8.9	1.8	1.0
Delayed modified training‐IRAP(2s)	6.2	1.8	1.1

#### Baseline training

With respect to the baseline training blocks, a Kruskal–Wallis test indicated a significant difference between the groups, *H*(3) = 32.78, *p* < .001. A post hoc Dunn test with *p* value adjusted for Bonferroni corrections indicated statistically significant differences between some groups: DMTS(2s) × Standard Training‐IRAP (*p* < .001), DMTS(2s) × Modified Training‐IRAP (*p* < .006), Standard Training‐IRAP × Delayed Modified Training‐IRAP(2s; *p* < .006). Overall, therefore, the results indicated that participants required significantly fewer blocks to reach criterion on the DMTS(2s) than on the standard training‐IRAP and modified training‐IRAP and significantly fewer on the delayed modified training‐IRAP(2s) relative to the standard training‐IRAP. No statistically significant difference was found between DMTS(2s) and delayed modified training‐IRAP(2s).

##### Mean number of AB, BC, CD, and DE baseline blocks

**FIGURE 6:**
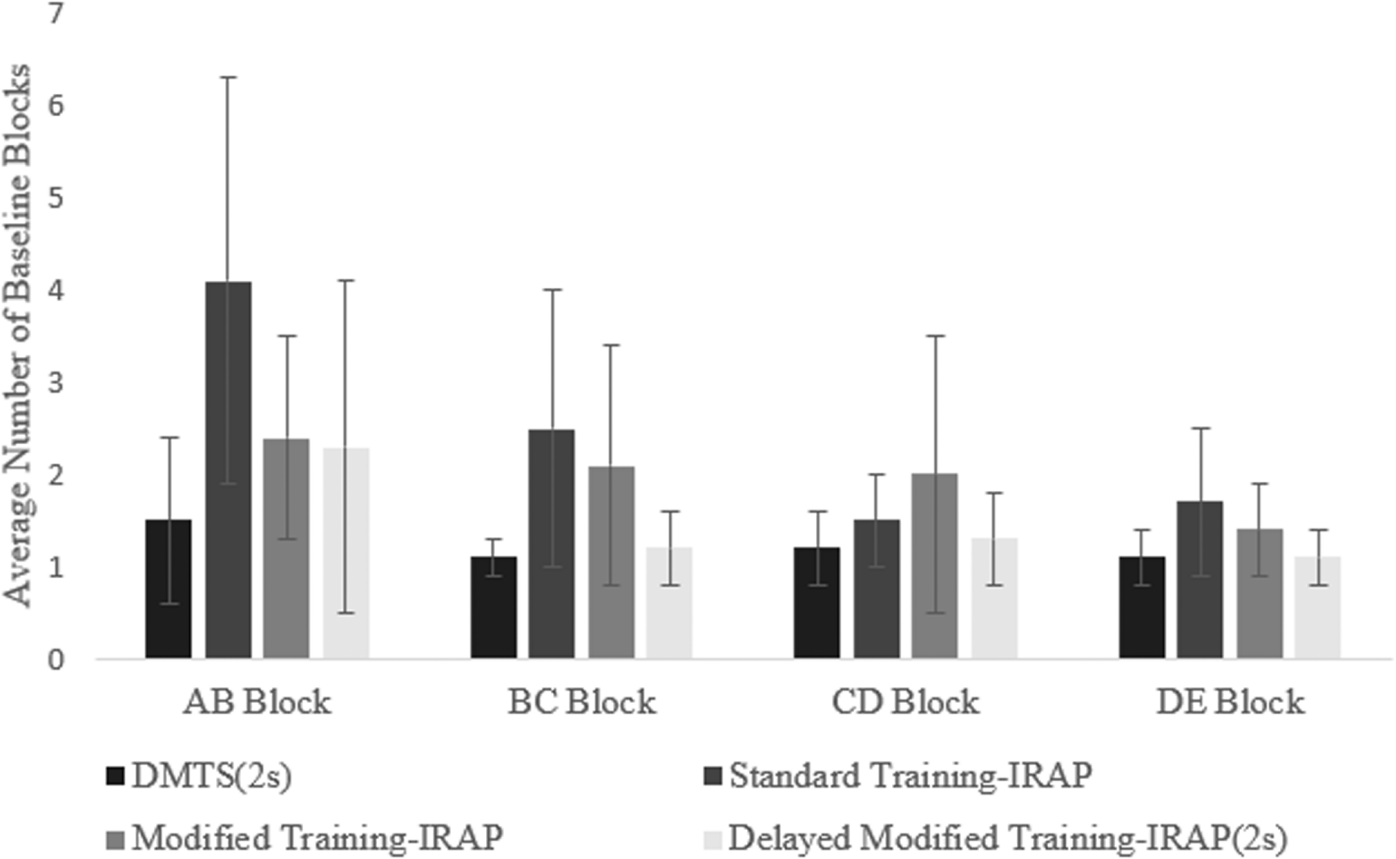
Average number of AB, BC, CD, and DE baseline blocks. Each bar represents the mean number of baseline blocks needed per procedure in AB, BC, CD and DE, along with their respective standard deviations.

The baseline blocks comprise the sequential presentation of the AB, BC, CD, and DE blocks. Figure 6 presents the mean number of AB, BC, CD, and DE baseline blocks, along with their respective standard deviations for each procedure.

*AB block*. A Kruskal–Wallis test indicated a significant difference between the groups, *H*(3) = 23.91, *p* < .001. With the Bonferroni‐corrected *p* value, statistically significant differences between some groups remained (according to a post hoc Dunn test): DMTS(2s) x Standard Training‐IRAP (*p* < .001), Standard Training‐IRAP x Delayed Modified Training‐IRAP(2s); (*p* = .012).

*BC Block*. A Kruskal–Wallis test indicated a significant difference between the groups, *H*(3) = 25.22, *p* < .001. With the Bonferroni‐corrected *p* value, statistically significant differences between some groups remained (according to a post hoc Dunn test): DMTS(2s) × Standard training‐IRAP (*p* < .001), DMTS(2s) × Modified Training‐IRAP (*p* = .006), Standard Training‐IRAP × Delayed Modified Training‐IRAP(2s; *p* = .024).

*CD Block*. A Kruskal–Wallis test indicated no significant difference between the groups, *H*(3) = 7.46, *p* = .06.

*DE Block*. A Kruskal–Wallis test indicated a significant difference between the groups, *H*(3) = 8.95, *p* = .03, but after the post hoc Dunn tests and adjusting the *p* value with the Bonferroni correction, no statistically significant differences between groups remained (all *p*s *≥* .05).

Overall, the results indicated that the differences between the mean number of baseline blocks per procedure were significant for only the initial two blocks (i.e., AB and BC); no significant difference was evident for the final two blocks (i.e., CD and DE). In the AB blocks, participants needed fewer blocks in the DMTS(2s) than in the standard training‐IRAP and fewer blocks in delayed modified training‐IRAP(2s) than in the standard training‐IRAP. In the BC blocks, participants needed fewer blocks in the DMTS(2s) than in the standard and modified training‐IRAP and fewer blocks in the delayed modified training‐IRAP(2s) than in the standard training‐IRAP.

#### Baseline mixed training

With respect to the baseline mixed training blocks, a Kruskal–Wallis test indicated a significant difference between the groups, *H*(3) = 14.95, *p* < .01. A post hoc Dunn test with Bonferroni correction indicated differences between two groups: DMTS(2s) and the standard training‐IRAP (*p* < .006). In general, therefore, the results indicated that participants required significantly fewer blocks to reach criterion in the DMTS(2s) only relative to the standard training‐IRAP.

#### Baseline mixed training without differential consequences

With respect to the baseline mixed training without differential consequences blocks, a Kruskal–Wallis test indicated differences between the groups, *H*(3) = 12.39, *p* < .01. With the Bonferroni‐corrected *p* value, post hoc Dunn tests indicated significant differences between: DMTS(2s) and standard training‐IRAP (*p* = .018) and standard training‐IRAP and modified training‐IRAP (*p* = .018). Overall, therefore, the results indicated that participants required significantly fewer blocks to reach criterion in only the DMTS(2s) relative to the standard training‐IRAP and in the modified training‐IRAP relative to the standard training‐IRAP.

### Response latency during probes for derived relations in standard training‐IRAP and modified training‐IRAP

Although no latency criterion was in place during probe blocks across any of the groups, the standard training‐IRAP involved a latency criterion for all other stages of the training procedure. Given how poor the yield was for the standard training‐IRAP group relative to the other groups, one possibility is that the reinforcement contingencies for fast responding in place during training gave rise to similarly fast responding in the absence of such reinforcement during the probe trials. To explore this possibility, a post hoc analysis was conducted to compare the participant's overall median response latencies during probe trials for the standard training‐IRAP and modified training‐IRAP. This exploratory comparison was conducted for these two procedures and not the others given the close structural similarities involved (i.e., simultaneous presentation of stimuli).^4^ To this end, a Mann–Whitney *U* test was conducted to test for a difference between the median response latencies of participants in the standard training‐IRAP (*M* = 1,986 ms, *Mdn* = 1,808 ms) versus modified training‐IRAP (*M* = 2,672 ms, *Mdn* = 2,400 ms). The result showed a statistically significant difference between the median response latencies of these groups (*U* = 6,912, *p* < .001). That is, participants responded significantly faster during the derived‐relations probes within the standard training‐IRAP procedure than they did when responding to the derived‐relations probes within the modified training‐IRAP procedure despite neither procedure requiring fast responding during this phase.

## DISCUSSION

The current study sought to compare the influence of DMTS(2s) and training‐IRAP procedures that were presented in three formats—standard training‐IRAP, modified training‐IRAP, and delayed modified training‐IRAP(2s)—on baseline acquisition and yield with respect to the emergence of two 5‐member equivalence classes. Regarding baseline acquisition, participants needed significantly fewer baseline training blocks when using the DMTS(2s) procedure than with both the standard training‐IRAP and modified training‐IRAP. However, no significant difference emerged between the DMTS(2s) and delayed modified training‐IRAP(2s) procedures. In terms of yield, the modified versions of the training‐IRAP consistently produced the highest yield across all criteria, whereas the standard training‐IRAP produced the lowest. The DMTS(2s) procedure performed above the standard training‐IRAP but below the modified training‐IRAP versions. Nonetheless, the statistical analyses did not support the superiority of modified versions of the training‐IRAP procedures over DMTS(2s) in terms of yield. Thus, the findings remain inconclusive in this respect. Future studies with larger samples are needed to clarify these results.

The results of the post hoc analysis may partly explain why the lowest yield was consistently observed for the standard training‐IRAP, at least when compared with the modified training‐IRAP at the strictest accuracy criterion. That is, the lowest yield may have been partly attributable to the reinforcement of rapid responses during baseline training. Following such reinforced responding, it is possible, if not likely, that this led to generalized fast responding during testing even when it was not a requirement, thus hindering accuracy. Therefore, one might ask whether adding an instruction before probe blocks specifying that there is no longer the need for fast responding would increase yield. Of course, it could be argued that other dimensions along which the procedures varied such as response topography, correction procedure, and the presentation of block feedback could also have contributed to the observed differences. In any case, future studies could systematically investigate this issue.

In contrast to the current findings, a number of other studies have reported the utility of the standard training‐IRAP for training and testing derived relations (e.g., Harte et al., 2018, 2020, 2021; Leech & Barnes‐Holmes, 2020; Leech et al., 2018). It should be noted, however, that the quantity of stimuli involved in these studies was notably lower than that employed currently. For example, Harte et al. (2020) trained A1B1C1 and A2B2C2 before testing for derived AC relations, whereas the current study involved a larger number of relations trained and tested as well as employing more stringent performance requirements. This suggests potential boundary conditions for use of the standard training‐IRAP to establish derived relations of varying complexity and may be worthy of further investigation.

The lack of statistically significant results for the modified versions of the training‐IRAP relative to DMTS(2s) raises doubts about whether increased experimental control of specific stimulus relations alone is sufficient to enhance yield. Specifically, the training‐IRAP format sought to control baseline relation stimulus‐control topographies (SCTs; Carrigan & Sidman, 1992) by specifying the relations between sample and comparison S+ and S− (e.g., A1/B1/Same‐Different and A1/B2/Same‐Different). Nevertheless, no statistically significant influence on yield emerged relative to the MTS training format. Other research has appealed to ensuring both SCTs for all baseline relations in explaining particularly high yield (e.g., Arantes & de Rose, 2015; de Rose et al., 2013; Grisante et al., 2014). However, all of these studies also included a pretraining session before the experimental phases during which participants underwent multiple‐exemplar training with additional stimuli presented in the same task structure. Perhaps this pretraining led to the improved learning observed in the experimental task (e.g., Nedelcu et al., 2015; Saunders & Spradlin, 1990, 1993). It could be useful for future work to systematically explore the influence of multiple‐exemplar training on yield in conjunction with comparisons of task training format.

An additional point concerning task format differences is that it may be possible to conceptualize the training‐IRAP format as involving a five‐term contingency relative to four terms for the MTS preparation. When conceptualizing the training‐IRAP in this way, one might consider the sample as the first term, the comparison the second, the response option the third, the response itself as the fourth term, and the feedback provided as the fifth term. This highlights a potentially important difference between the four terms arguably involved in the MTS preparation (sample‐comparison‐response‐feedback). On balance, some authors have argued that the topography of “choosing” is not itself a response in functional terms, a consideration perhaps necessary for such an analysis (Hayes & Barnes, 1997). With respect to responding in both procedures, those same authors argued that the correct comparison itself goes with the class on an MTS procedure but on a training‐IRAP and related formats^5^ the response option is rather seen as a relational cue that describes how the stimuli go together (see Hayes & Barnes, for a full explanation of this issue). Indeed, investigating these issues experimentally going forward seems a worthy endeavor. For example, imagine an experiment whereby no overt response is required during the task (e.g., respondent‐type training) and the presentation of one arbitrary stimulus is consistently followed by the presence and subsequent withdrawal of another. Previous studies of this type reported that when such stimulus sequences were later employed in a typical MTS, both adults and children as young as 5 years of age responded in accordance with coordination (i.e., sameness) relations (e.g., Barnes et al., 1996; Leader et al., 1996; Smeets et al., 1997). Extending such investigations to the training‐IRAP format could help determine whether participants would respond with “Same” to pairs of stimuli that consistently followed one another and with “Different” to those that did not. This may provide a means to explore the functional rather than purely topographical variables at play in symbolic learning.

Another interesting finding was that the addition of the observational response and 2‐s delay components to the modified training‐IRAP seemed to contribute to an increased efficiency in baseline relation training to near‐DMTS(2s) levels. That is, considering the standard training‐IRAP and its modified versions, there was a significant reduction in the mean number of baseline relation training blocks required by participants from the standard training‐IRAP (required the most baseline training blocks) to the delayed modified training‐IRAP (2s; required the fewest baseline training blocks). Interestingly, however, no significant difference in this respect emerged between the modified and delayed modified training‐IRAP(2s), a finding that is consistent with MTS‐based findings that delay alone has no significant influence on baseline acquisition (Arntzen et al., 2018; Lian & Arntzen, 2013). Furthermore, although a significant difference in yield was observed between the standard training‐IRAP and the delayed modified training‐IRAP(2s), introduction of these components did not seem to improve yield between the modified training‐IRAP and delayed modified training‐IRAP(2s). The latter finding may be seen as being somewhat at odds with the wider MTS literature in which yield often improves as a function of increasing presentation delays during training (Arntzen, 2006). In any case, replicating the current findings with larger samples of participants seems important, as does exploring the influence of different delays within the training‐IRAP format on yield (e.g., 4, 6, and 12‐s delays; e.g., Arntzen, 2006; Arntzen et al., 2018; Lian & Arntzen, 2013).

A procedural issue that may be worthy of further attention pertains to the instructions provided to MTS and training‐IRAP participants as well as the use of familiar (preexperimental) words—“Same” and Different”—as response options in the latter. The current use of both of these variables was consistent with that in the published literature employing both procedures (e.g., Ayres‐Pereira & Arntzen, 2021; Boldrin et al., 2022; Harte et al., 2021; Leech & Barnes‐Holmes, 2020), and the procedures were broadly similar in that they instructed participants about the format of stimulus presentation and asked them to “discover the relationship/relation” between the stimuli. However, the fact remains that necessary differences in the descriptions of how stimuli were presented were also involved. For example, an instruction of the sort, “An image will be shown . . . followed by another one,” may foster particular discriminations between the sample and comparison stimuli. In addition, instructions of the sort, “discover the relationship,” may facilitate the establishment of experimenter‐defined stimulus control. A related consideration involves the use of the preexperimental words “Same” and “Different” within the training‐IRAP variations. One might argue it preferable to first establish their stimulus control experimentally using arbitrary symbols (e.g., via an initial nonarbitrary training phase) and then employ these experimentally established cues in the procedure. Future studies may seek to control for and/or directly target some of these variables in progressing the current line of research.

It should also be noted that other studies have explored alternatives to MTS for equivalence class formation. These include go/no‐go trial presentations (e.g., Debert et al., 2007; Lantaya et al., 2018; Zhelezoglo et al., 2021), the SPYN procedure (e.g., Gallant et al., 2021; Hensel et al., 2024), and relational tacting and listener training (e.g., Cordeiro et al., 2021). There are many procedural differences involved in each of these preparations, each of which may or may not increase the likelihood of equivalence class formation. For example, the “no‐go” element involved in the go/no‐go procedure is a feature not included in either MTS or training‐IRAP formats. To what extent may the inclusion or omission of this procedural element increase yield compared to, for example, delays in stimulus presentation and the incorporation of an observational response as in the current study? As another example, consider that DMTS(2s) and delayed modified training‐IRAP(2s) differ from the standard and modified training‐IRAPs in the successive versus simultaneous presentation of sample‐comparison stimuli. Some studies employing MTS alternatives (e.g., go/no‐go) involving compound stimuli have explored the influence of this presentation format on discrimination and equivalence class formation in adults (e.g., Debert et al., 2007; Grisante et al., 2013) and children with learning difficulties (e.g., Silva & Debert, 2017). Some of this work suggests that although symmetry is readily demonstrated in both populations with compound stimulus presentation, equivalence is not. To what extent do these or similar findings apply to these populations using the current procedural variations? Although a detailed consideration of these details is beyond the scope of the current article, conducting systematic comparisons between and among these procedures could provide valuable experimental and conceptual insights, particularly if extending the current work to use with other populations (e.g., children with learning disabilities).

Finally, it may also be useful to briefly consider other areas within the study of derived relations that connect with the current work beyond a focus on yield alone. For example, other research has explored the influence of different MTS procedures on subsequent transfer of stimulus functions (e.g., simultaneous MTS vs. DMTS; Bortoloti & de Rose, 2009, 2012), generally finding that greater functional transfer is observed with DMTS (Bortoloti & de Rose, 2009, 2012). Indeed, most research on function transfer to date has relied largely on MTS (e.g., Bortoloti et al., 2019; Gomes et al., 2019; Perez, de Almeida, et al., 2019; Schmidt et al., 2021), with only a handful thus far employing the standard training‐IRAP in this respect (Leech & Barnes‐Holmes, 2020; Leech et al., 2018). Therefore, it may be useful, to explore any differences on subsequent function transfer when establishing derived relations with MTS versus training‐IRAP variations. Such explorations and the other suggestions for future research described above stand to contribute to understanding how subtleties in learning history differentially affect the establishment of derived relations.

## AUTHOR CONTRIBUTIONS

MHS: Conceptualization, funding acquisition, methodology, software, investigation, data curation, visualization, original draft manuscript, review, and revisions. CH: Conceptualization, methodology, manuscript review and revisions. DAP: Formal analysis, validation, manuscript review and revisions. JCR: Conceptualization, funding acquisition, methodology, supervision, resources, original draft manuscript.

## CONFLICT OF INTEREST STATEMENT

The authors declare no conflicts of interest.

## ETHICS APPROVAL

The study was approved by the Human Studies Review Board of the Universidade Federal de São Carlos (CAAE: 56710222.0.0000.5504).

## Data Availability

All data supporting the findings of this study are included in the article and its appendix.

## Footnotes

^1^
The reader might initially liken the training‐IRAP to the stimulus‐pairing‐yes‐no (SPYN) procedure (see Gallant et al., 2021; Hensel et al., 2024). And indeed, there certainly is overlap. However, although the training format of the methodologies shares certain characteristics, the response options employed (Yes/No vs. Same/Different) can arguably yield distinct experimental procedures. For example, in the absence of specific instructions before an experiment, if asked “A1B1—Same or Different,” a verbally able participant presumably understands that they need to identify the relation between the stimulus pair. Conversely, when asked “A1B1—Yes or No,” without further instruction (e.g., if they “go together” respond YES; if they do not, respond NO), participants may struggle to respond YES or NO to the stimulus pairs or respond differentially across pairs without additional guidance (i.e., YES or NO on what basis?). According to the RFT literature, responding to Same/Different versus Yes/No may be considered functionally distinct, involving different learning histories. Specifically, RFT posits that “Same” and “Different” function as cues that indicate the pattern of relation between stimuli, whereas Yes and No serve as relational coherence indicators (RCIs; see Hayes & Barnes, 1997, for a relevant discussion on relational coherence), with RCIs only applicable once base levels relational cues have been established (see Maloney & Barnes‐Holmes, 2016; Murphy et al., 2022, for experimental evidence for this distinction).

^2^

*Node* refers to a stimulus that is related in baseline training to two or more stimuli. For example, in a linear series ABC training, B is considered a node, as it was related to A and C during training. Thus, the probed relation CA has one node (Fields et al., 1984).

^3^
In the RFT literature, the stimulus presented at the top of the screen within an IRAP is referred to as the label stimulus and the stimulus in the center of the screen is referred to as the target stimulus. However, to facilitate the comparison of trial format between MTS and training‐IRAP procedures, the terms “label” and “target” were substituted for those more commonly employed in the MTS literature: sample stimulus and comparison stimulus, respectively.

^4^
Comparing the standard training and modified training‐IRAP groups with others involving nonsimultaneous or delayed stimulus presentations was not deemed feasible due to differences in the response windows involved. To clarify, in the simultaneous presentation format, all four stimuli (i.e., sample, comparison, and two response options) are present on the screen during the response window. In the nonsimultaneous format, however, in the DMTS(2s) group the corresponding response window is present only after participants have made an observational response to the sample, followed by its withdrawal and a 2‐s delay before the stimulus comparisons are presented. The response window then occurs during the presentation of the two stimulus comparisons. In the delayed modified training‐IRAP(2s) group, the response window is available only after participants have emitted an observational response to the sample, followed by its withdrawal and a 2‐s delay before the stimulus comparison is presented. After emitting an observational response to the comparison, the response options are presented and the response window is available with the stimulus comparison remaining on the computer screen. Given these response window differences as well as the number of stimuli participants needed to attend to, meaningful response latency comparisons were deemed feasible between only the standard training‐IRAP and modified training‐IRAP.

^5^
The reader should note that the modifications yielding the modified training‐IRAP resulted in a procedure that is somewhat similar to what has been termed the relational evaluation procedure (REP; a method designed for participants to report or evaluate stimulus relations; see, e.g., Barnes‐Holmes et al., 2001; Hayes et al., 2016). This may not be surprising given that the IRAP itself was developed, in part, from the REP (see Barnes‐Holmes & Harte, 2022b, for further details). Given that the current study began with the standard training‐IRAP and involved making modifications to that format specifically, we have found it more accurate and appropriate to refer to the resulting procedures as modified versions of the training‐IRAP rather than REPs. Furthermore, the REP refers to a wide range of performances and procedures (e.g., Hayes et al., 2016; cf. Stewart et al., 2004), whereas the training‐IRAP generally refers to a more uniform training preparation (e.g., Harte et al., 2021; cf. Leech et al., 2018). Nonetheless, it is important to recognize that the modified versions of the training‐IRAP involved in the current study may be seen as REP‐like, and thus the resulting implications may also be relevant for the use of the REP.

## APPENDIX A

**TABLE A1:** Number of correct responses in the probe of derived relations for each procedure.

	EA	Baseline	DA	Baseline	EB	Baseline	CA	Baseline	DB
DMTS(2s)									
P01	22/24	16/16	20/24	16/16	5/24	15/16	22/24	15/16	20/24
P02	24/24	16/16	24/24	16/16	24/24	16/16	23/24	16/16	24/24
P03	0/24	16/16	24/24	16/16	24/24	16/16	1/24	16/16	0/24
P04	0/24	15/16	18/24	15/16	1/24	16/16	1/24	15/16	0/24
P05	24/24	16/16	23/24	16/16	24/24	16/16	24/24	16/16	24/24
P06	24/24	16/16	24/24	16/16	24/24	16/16	23/24	16/16	24/24
P07	24/24	15/16	24/24	16/16	24/24	16/16	24/24	16/16	24/24
P08	24/24	16/16	23/24	15/16	24/24	16/16	24/24	15/16	24/24
P09	24/24	16/16	24/24	16/16	24/24	16/16	24/24	16/16	24/24
P10	0/24	16/16	24/24	15/16	2/24	16/16	1/24	16/16	24/24
P11	2/24	16/16	23/24	16/16	24/24	16/16	1/24	14/16	0/24
P12	24/24	15/16	1/24	16/16	6/24	16/16	24/24	15/16	23/24
P13	24/24	16/16	23/24	16/16	22/24	16/16	21/24	16/16	23/24
P14	24/24	16/16	24/24	15/16	23/24	16/16	24/24	16/16	24/24
P15	24/24	16/16	24/24	16/16	24/24	16/16	24/24	16/16	24/24
P16	24/24	15/16	24/24	15/16	24/24	16/16	23/24	15/16	23/24
P17	0/24	16/16	24/24	16/16	24/24	16/16	0/24	16/16	1/24
Standard training‐IRAP									
P01	18/24	16/16	12/24	16/16	12/24	16/16	17/24	15/16	16/24
P02	0/24	15/16	2/24	16/16	1/24	15/16	24/24	15/16	7/24
P03	24/24	16/16	23/24	16/16	24/24	15/16	24/24	14/16	21/24
P04	2/24	16/16	2/24	16/16	22/24	15/16	8/24	15/16	23/24
P05	12/24	16/16	12/24	14/16	12/24	14/16	12/24	15/16	19/24
P06	24/24	16/16	24/24	15/16	24/24	16/16	24/24	16/16	24/24
P07	18/24	16/16	22/24	16/16	21/24	15/16	20/24	14/16	24/24
P08	23/24	16/16	24/24	16/16	24/24	16/16	20/24	16/16	24/24
P09	23/24	15/16	21/24	16/16	23/24	15/16	23/24	15/16	24/24
P10	16/24	14/16	17/24	16/16	21/24	15/16	12/24	16/16	24/24
P11	24/24	16/16	24/24	16/16	24/24	16/16	23/24	16/16	23/24
P12	9/24	16/16	10/24	16/16	20/24	16/16	12/24	16/16	10/24
P13	10/24	15/16	9/24	14/16	15/24	14/16	12/24	15/16	9/24
P14	21/24	16/16	22/24	16/16	24/24	16/16	24/24	16/16	24/24
P15	21/24	16/16	23/24	15/16	24/24	15/16	22/24	16/16	24/24
P16	24/24	16/16	24/24	16/16	24/24	16/16	24/24	16/16	24/24
P17	6/24	16/16	17/24	16/16	15/24	15/16	14/24	16/16	21/24
Modified training‐IRAP									
P01	21/24	16/16	24/24	16/16	23/24	16/16	24/24	16/16	24/24
P02	24/24	16/16	24/24	16/16	24/24	16/16	23/24	16/16	24/24
P03	24/24	16/16	24/24	16/16	24/24	16/16	24/24	16/16	24/24
P04	24/24	16/16	23/24	16/16	23/24	16/16	23/24	16/16	24/24
P05	20/24	15/16	24/24	16/16	22/24	16/16	22/24	16/16	23/24
P06	23/24	16/16	24/24	14/16	23/24	16/16	24/24	16/16	24/24
P07	22/24	16/16	24/24	16/16	23/24	16/16	22/24	16/16	24/24
P08	12/24	16/16	12/24	16/16	12/24	16/16	12/24	15/16	12/24
P09	7/24	15/16	12/24	16/16	16/24	16/16	20/24	15/16	22/24
P10	15/24	15/16	2/24	16/16	24/24	16/16	24/24	16/16	23/24
P11	23/24	16/16	23/24	14/16	23/24	15/16	24/24	16/16	12/24
P12	24/24	16/16	24/24	16/16	24/24	16/16	24/24	16/16	24/24
P13	24/24	16/16	24/24	16/16	24/24	16/16	24/24	16/16	24/24
P14	24/24	16/16	24/24	16/16	24/24	16/16	24/24	16/16	24/24
P15	24/24	16/16	23/24	16/16	23/24	16/16	24/24	16/16	23/24
P16	24/24	16/16	24/24	16/16	24/24	16/16	24/24	16/16	24/24
P17	23/24	16/16	23/24	16/16	24/24	16/16	24/24	16/16	24/24
Delayed modified training‐IRAP(2s)									
P01	16/24	16/16	10/24	16/16	9/24	16/16	18/24	15/16	24/24
P02	24/24	16/16	24/24	16/16	24/24	16/16	24/24	16/16	23/24
P03	24/24	16/16	24/24	16/16	24/24	16/16	24/24	16/16	24/24
P04	10/24	14/16	18/24	15/16	17/24	15/16	21/24	16/16	4/24
P05	24/24	15/16	23/24	15/16	23/24	16/16	24/24	14/16	24/24
P06	24/24	16/16	23/24	16/16	24/24	16/16	24/24	16/16	24/24
P07	23/24	16/16	24/24	16/16	24/24	16/16	24/24	16/16	24/24
P08	24/24	16/16	24/24	16/16	23/24	16/16	24/24	16/16	24/24
P09	23/24	15/16	23/24	16/16	21/24	16/16	19/24	16/16	24/24
P10	2/24	16/16	24/24	16/16	24/24	16/16	0/24	16/16	24/24
P11	23/24	16/16	24/24	16/16	24/24	16/16	24/24	15/16	24/24
P12	19/24	16/16	24/24	16/16	24/24	16/16	24/24	15/16	24/24
P13	24/24	16/16	23/24	16/16	24/24	16/16	24/24	16/16	24/24
P14	24/24	16/16	24/24	16/16	24/24	16/16	24/24	16/16	24/24
P15	24/24	16/16	24/24	16/16	24/24	16/16	24/24	16/16	24/24
P16	24/24	16/16	23/24	16/16	24/24	16/16	24/24	16/16	23/24
P17	24/24	16/16	24/24	16/16	24/24	16/16	24/24	16/16	24/24

## APPENDIX B

### Number of training blocks

The tables present individual participant data for the number of training blocks in DMTS (2s; Table B1), standard training‐IRAP (Table B2), modified training‐IRAP (Table B3), and delayed modified training‐IRAP(2s; Table B4).

**TABLE B1:** Number of training blocks in DMTS(2s) for each participant.

	Baseline training				Baseline mixed training	Baseline mixed training without differential consequences
	AB	BC	CD	DE		
P01	4	1	1	1	2	1
P02	1	1	2	1	1	1
P03	1	1	1	1	1	1
P04	2	1	1	1	2	1
P05	1	1	1	2	1	1
P06	1	1	2	1	1	1
P07	1	1	1	1	1	1
P08	1	1	2	1	1	1
P09	1	1	1	1	1	1
P10	1	1	1	1	1	1
P11	3	1	1	1	2	1
P12	2	2	1	2	1	1
P13	1	1	1	1	1	1
P14	2	1	1	1	1	1
P15	1	1	1	1	2	1
P16	1	1	1	1	1	1
P17	1	1	1	1	1	1

**TABLE B2:** Number of training blocks in standard training‐IRAP for each participant.

	Baseline training				Baseline mixed training	Baseline mixed training without differential consequences
	AB	BC	CD	DE		
P01	2	3	2	3	2	2
P02	3	1	2	2	2	1
P03	6	4	3	4	4	1
P04	4	2	1	1	3	1
P05	3	9	3	3	4	2
P06	4	2	1	1	3	4
P07	10	4	3	3	5	1
P08	4	2	2	1	1	1
P09	5	2	1	1	2	1
P10	4	2	1	3	1	1
P11	3	8	2	2	4	2
P12	5	3	2	2	5	1
P13	2	3	2	2	3	1
P14	3	1	2	1	2	1
P15	7	2	1	2	2	1
P16	2	1	1	1	2	1
P17	8	2	2	1	1	3

**TABLE B3:** Number of training blocks in modified training‐IRAP for each participant.

	Baseline training				Baseline mixed training	Baseline mixed training without differential consequences
	AB	BC	CD	DE		
P01	5	3	1	2	2	1
P02	4	6	7	2	1	1
P03	4	1	2	1	1	1
P04	3	1	2	2	1	1
P05	3	4	4	3	2	1
P06	3	1	1	1	1	1
P07	3	2	2	2	2	1
P08	4	3	4	2	3	1
P09	2	3	1	1	3	1
P10	2	1	4	1	2	1
P11	3	5	2	3	4	1
P12	2	2	1	1	1	1
P13	2	2	1	1	1	1
P14	1	1	1	1	1	1
P15	2	2	3	2	4	1
P16	1	1	1	1	1	1
P17	2	2	2	1	1	1

**TABLE B4:** Number of training blocks in delayed modified training‐IRAP(2s) for each participant.

	Baseline training				Baseline mixed training	Baseline mixed training without differential consequences
	AB	BC	CD	DE		
P01	8	1	1	1	2	1
P02	2	1	1	2	1	1
P03	2	1	1	1	1	1
P04	5	2	2	2	2	1
P05	2	1	1	1	2	1
P06	3	2	2	2	5	1
P07	1	2	1	1	1	1
P08	1	1	1	1	1	1
P09	3	1	1	1	2	1
P10	2	1	2	1	3	1
P11	1	1	2	1	1	1
P12	2	1	1	1	1	1
P13	3	2	2	1	1	2
P14	1	1	1	1	1	1
P15	1	1	2	1	1	1
P16	2	1	1	1	2	1
P17	1	2	1	1	3	1
